# Circadian rhythm types and shift work demands shape sleep quality and depressive symptoms in shift-working nurses

**DOI:** 10.3389/fpubh.2025.1667778

**Published:** 2025-09-23

**Authors:** Huihan Zhao, Qiuxia Li, Huiqiao Huang, Feihong Lan, Huijuan Yang, Yu He, Zhaoquan Huang

**Affiliations:** ^1^School of Information and Management, Guangxi Medical University, Nanning, Guangxi, China; ^2^Department of Hematology, The First Affiliated Hospital of Guangxi Medical University, Nanning, Guangxi, China; ^3^University Engineering Research Center of Digital Medicine and Healthcare, Guangxi Medical University, Nanning, Guangxi, China; ^4^Department of Health Management and Division of Physical Examination, The First Affiliated Hospital of Guangxi Medical University, Nanning, Guangxi, China; ^5^Department of Nursing, The Second Affiliated Hospital of Guangxi Medical University, Nanning, Guangxi, China; ^6^Department of Nursing, The People’s Hospital of Du’an Yao Autonomous County, Hechi, Guangxi, China; ^7^Department of Clinical Laboratory, The First Affiliated Hospital of Guangxi Medical University, Nanning, Guangxi, China; ^8^Department of Pathology, The First Affiliated Hospital of Guangxi Medical University, Nanning, Guangxi, China

**Keywords:** circadian rhythm, work demand, sleep, depression, shift nurses

## Abstract

**Objective:**

To examine the predictive, moderating, and combined effects of circadian rhythm types and shift work demands on sleep quality and depressive symptoms among shift-working nurses.

**Methods:**

A cross-sectional study was conducted between May 1, 2024, and May 31, 2025. Shift-working nurses were recruited using convenience sampling at a tertiary hospital in Guangxi, China. Circadian rhythm types, sleep quality and depressive symptoms were assessed using the Circadian Type Inventory [CTI; including flexibility–rigidity (FR) and languidness–vigorousness (LV)], the Pittsburgh Sleep Quality Index (PSQI) and the Patient Health Questionnaire-9 (PHQ-9). Objective data on shift work demands over a four-week period were extracted from the hospital nursing management system, including number of night shifts, total shift hours, and shift workload exposure. Generalized linear modeling (GLM), nonlinear curve fitting, and Monte Carlo simulation were used for data analysis.

**Results:**

A total of 288 shift nurses were included. The GLMs showed that depressive symptoms (*β* = 0.245), languidness (*β* = 0.065), shift work hours (*β* = 0.093), and body mass index (*β* = −0.056) were significant predictors of poorer sleep quality. Poorer sleep quality (*β* = 0.314), flexibility (*β* = −0.129), languidness (*β* = 0.159), and the interaction between sleep quality and flexibility (*β* = 0.091), between languidness and shift work hours (*β* = 0.069) significantly predicted depressive symptoms. Nonlinear analysis identified a potential threshold effect, with more than 24 shift work hours in 4 weeks linked to poorer sleep quality. Dynamic simulations demonstrated that the combined effects of circadian rhythm types and shift work demands corresponded to distinct dose–response patterns in sleep quality and depressive symptoms.

**Conclusion:**

Circadian rhythm types and shift work demands jointly shape sleep quality and depressive symptoms in shift nurses, with distinct dose–response patterns. These findings highlight the importance of circadian-informed shift scheduling to improve sleep and mental health among shift nurses.

## Graphical abstract

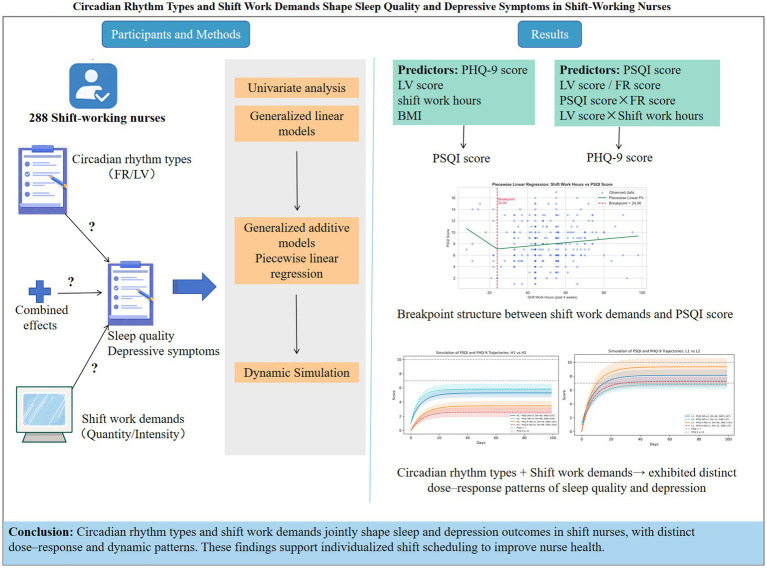

## 1 Introduction

To meet the growing demands of globalisation and a 24-h society, atypical work schedules have become increasingly common, involving 14% ~ 38% of the global workforce (1–3). Shift work disrupts sleep–wake patterns and often results in chronic sleep deprivation, contributing to circadian rhythm disruption (4). Circadian rhythms regulate a wide range of physiological and behavioral processes (5), which undoubtedly contribute to an increased risk of various pathological conditions.

Given the increasing demand for medical services and the need of uninterrupted patient care, nurses are required to work around-the-clock shifts. The negative impacts of shift work on nurses have been well documented (6, 7). Studies show that nearly 20% of night-shift nurses suffer from shift work sleep disorders (8), and 41.2–60.4% experience poor sleep quality (9). Moreover, a multicenter study in China found that 58.82% of shift nurses exhibited depressive symptoms, and 62.08% of them experienced anxiety symptoms (10). Sleep and depression problems are prevalent among shift nurses. Moreover, workers with insufficient or poor-quality sleep, especially under shift work schedules, are at higher risk of depression (11). Evidence from a multicenter study shows that nurses with adequate sleep experience lower stress levels (12). Poor sleep not only impairs nurses’ physical and mental health but also compromises job performance, with ripple effects on patient safety (13–15). However, the predictors and moderators of sleep and depression among shift nurses remain underexplored, posing a challenge for clinical nursing management.

The Job Demands-Resources (JD-R) theory provides a valuable framework for understanding how shift work demands influence the sleep and psychological well-being of shift nurses. As a job design theory, the JD-R model (16, 17) explains how job demands and resources interact to shape employee well-being and performance. Job demands refer to aspects of work that require sustained physical and mental effort and are associated with physiological and/or psychological costs as well as health impairment processes (17, 18). Shift nurses face sustained and intense work demands, such as repeatedly irregular night shifts and complex patient care, which can deplete physical and emotional resources, lead to sleep debt, and trigger adverse health outcomes. A nationwide cross-sectional study also demonstrated a strong interconnectedness between job demands, resources, and depressive symptoms in critical care nurses (19). Empirical studies also support that repeated high work demands are linked to increased risks of sleep disturbances, depression, and anxiety among shift nurses (10, 20–23). However, most existing studies have relied on self-reports. Objective and real-world assessments of shift work demands remain limited, yet they are crucial for clarifying health impacts and guiding effective interventions for shift nurses.

Job resources are the physical, psychological, social, or organizational aspects of work that help employees achieve goals and alleviate the health-damaging consequences (17, 18). The JD-R theory further posits a reciprocal relationship between job and personal resources (17), suggesting that individuals with higher levels of personal resources—such as resilience and adaptability—are better able to buffer the negative impact of shift work demands. With increasing recognition of individual endogenous differences, the concept of circadian rhythm types has been introduced as a form of personal resources, reflecting individual variations in adaptability and tolerance to shift work. Circadian rhythm types comprise two dimensions: stability (flexible–rigid) and amplitude (languid–vigorous) (24). These circadian traits have been shown to be associated with sleep-related outcomes and other health risks in shift workers (25–27). Research findings and expert consensus consistently suggest that individual circadian profiles should be taken into account when developing occupational health policies, interventions, and shift scheduling strategies (28–30). However, the independent and combined effects of circadian rhythm types and shift work demand on nurses’ health outcomes remain insufficiently explored.

Taken together, this study aimed to integrate objective work demands data with self-reported circadian types: first, to identify key predictors and moderators of sleep quality and depressive symptoms; second, to examine their combined effects and potential dose–response relationships. The findings are expected to provide important evidence for developing individualized shift scheduling strategies and health promotion interventions for shift-working nurses.

## 2 Methods

### 2.1 Study design and participants

This cross-sectional study was conducted between May 1, 2024, and May 31, 2025, at a tertiary general teaching hospital in Guangxi, China. Shift-working nurses were recruited using convenience sampling during routine physical examinations at the hospital’s health examination center. Written informed consent was obtained from all participants. Inclusion criteria were as follows: (1) age 20–45 years, chosen to minimize potential confounding from menopause-related hormonal changes in older female nurses and work schedule adjustments; (2) full-time registered nurses; (3) ≥6 months of clinical experience; and (4) having worked in a fixed ward and experienced at least one rotating shift per month over the past 6 months. Exclusion criteria included: (1) having experienced major illness (e.g., cancer, cardiovascular or cerebrovascular disease, diabetes), major surgical procedures, major psychiatric disorders (e.g., schizophrenia, bipolar disorder), sickness absence (≥3 days), or major family life events (e.g., bereavement or marital disruption) within the past 6 months; (2) pregnancy or breastfeeding within the past 6 months; (3) travel across time zones within the past month; or (4) a lack of available shift work data.

### 2.2 Measures

Eligible participants were invited to complete a paper-based self-report questionnaire designed to collect general information (including gender, age, ethnicity, marital status, number of children, education level, years of work experience, professional title, years of shift work experience, and shift model), and to assess circadian rhythm types, sleep quality, and depressive symptoms using the Circadian Type Inventory (CTI), the Pittsburgh Sleep Quality Index (PSQI), and the Patient Health Questionnaire-9 (PHQ-9), respectively. The principal investigators (ZH and QL) administered the questionnaires on-site and verified their completeness immediately upon collection.

#### 2.2.1 Circadian rhythm types

The original CTI was developed by Folkard et al. (24) and subsequently revised by Di Milia et al. (31) to assess individual adaptability to shift work. The Chinese version CTI, translated and validated by Qi et al. (32), has demonstrated good psychometric properties (Cronbach’s *α* = 0.80 for FR; 0.73 for LV). The CTI assesses the stability and amplitude of circadian rhythms across two dimensions: rhythm stability (flexible–rigid, FR; 5 items) and rhythm amplitude (languid–vigorous, LV; 6 items). Items are rated on a 5-point Likert scale (1 = “almost never” to 5 = “almost always”). Higher FR scores indicate greater flexibility in adapting sleep–wake patterns, while higher LV scores reflect greater languidness and greater vulnerability to drowsiness and sleep loss. In this study, the CTI showed acceptable internal consistency (Cronbach’s *α* = 0.843 for FR; 0.719 for LV).

#### 2.2.2 Sleep quality

The PSQI was originally developed by Buysse et al. (33) to assess sleep quality over the past month. The Chinese version of the PSQI also demonstrated good internal consistency with Cronbach’s *α* ranging from 0.82 to 0.83 (34). A total PSQI score >7 has been validated as an effective cutoff to distinguish individuals with insomnia from the general population, with a sensitivity of 98.3% and a specificity of 90.2% (35). The PSQI includes 19 items covering seven components: subjective sleep quality, sleep latency, sleep duration, sleep efficiency, sleep disturbances, use of sleep medications, and daytime dysfunction. Each component is scored from 0 to 3, yielding a total score ranging from 0 to 21, with higher scores indicating poorer sleep quality and a cutoff score >7 indicating positive screening for insomnia. In this study, the PSQI showed acceptable internal consistency (Cronbach’s *α* = 0.748).

#### 2.2.3 Depressive symptoms

The PHQ-9, developed by Kroenke et al. (36), has been validated in Chinese populations by Zhang et al. (37), demonstrating good internal consistency (Cronbach’s *α* = 0.854), and is a widely used self-report tool for assessing the severity of depressive symptoms. A cutoff score of ≥10 is widely recognized as the optimal threshold for probable depression, offering good sensitivity and specificity (both 0.85) (38). The PHQ-9 consists of 9 items rated on a 4-point Likert scale (0 = “not at all” to 3 = “nearly every day”), yielding a total score between 0 and 27. Higher scores reflect greater levels of depressive symptomatology, with a cutoff score ≥10 indicating a positive screen for depressive symptoms. In this study, the PHQ-9 was used to assess depressive symptoms over the past month and demonstrated good internal consistency (Cronbach’s *α* = 0.871).

#### 2.2.4 Work demands

Work demands over the past 4 weeks were assessed across two dimensions: work quantity and work intensity, based on data extracted from the hospital’s nursing management system.

- *Work quantity* was measured using the following indicators: total evening shift count, total night shift count, total shift count, shift work hours, day work hours and total work hours. Total shift count was the sum of evening and night shifts. Shift work hours represented the cumulative hours worked during evening and night shifts. Total work hours represented the cumulative hours worked across both day and shift work. Shift work was defined as work performed between 18:00 and 08:00. An evening shift was defined as at least four consecutive working hours between 18:00 and 00:00, while a night shift was at least four consecutive hours between 00:00 and 08:00. A long night shift (18:00 to 08:00 with a three-hour nap break) was also classified as a night shift.
- *Work intensity* reflected patient care demands and was measured using workload, and workload exposure, calculated separately for day and shift work over the four-week observation period. Workload was defined as the ratio of expected to actual nurse-to-patient ratios (NPRs) (39, 40). The actual NPR for day and shift work during the observation period was calculated as the ratio of active primary nurses to assigned patients, and extracted from the hospital’s nursing management system. The expected NPR, representing the ideal nurse-to-patient ratio based on patient care needs at the ward level, was calculated corresponding to each participant’s unit during the observation period. Expected NPR was derived from patient severity scores proposed by Welton et al. (40), where mild (score = 0), moderate (score = 1), and severe (score = 2) conditions corresponded to expected NPRs of 1:8, 1:3, and 1:1, respectively. Considering the exponential relationship between patient severity and expected NPR, this study fitted the following exponential function (39): Expected NPR = 0.1154 × exp. (1.0791 × patient severity). The case mix index (CMI) was used as a proxy for patient severity, clinical complexity, and resource consumption, with higher CMI values indicating more severe conditions and greater care demands (41). Unit-level CMI data during the observation period were extracted from the China Healthcare Security Diagnosis-Related Groups (CHS-DRGs) system and normalized to a 0 ~ 2 scale for the calculation of expected NPR. Workload exposure for day and shift work was calculated as: Day/shift workload exposure = workload × corresponding work hours.

### 2.3 Statistical analysis

All data were analyzed using Python and relevant statistical packages. Due to missing inpatient and NPR data, participants from the emergency department (*n* = 7), anesthesiology department (*n* = 1), and hemodialysis unit (*n* = 2) were excluded from work intensity analysis. A two-tailed *p*-value <0.05 was considered statistically significant in this study.

(1) *Descriptive statistics*: Continuous variables were summarized as mean ± standard deviation (SD) (normal distribution) or median with interquartile range (IQR) (skewed distribution). Categorical variables were reported as frequencies and percentages.
(2) *Univariate analysis*: Group differences in PSQI, PHQ-9, FR, and LV scores across participant characteristics were assessed using the Mann–Whitney U test or Kruskal–Wallis H test. Spearman’s rank correlation analysis was performed to assess associations among PSQI scores, PHQ-9 scores, FR/LV scores, work demand variables, and other continuous variables.
(3) *Generalized Linear Modeling*: Generalized linear models (GLMs) were constructed to predict both PSQI and PHQ-9 scores. All variables entered into the GLMs exhibited skewed distributions and were therefore preprocessed by removing outliers and handling missing values, followed by Yeo–Johnson transformation and *z*-score standardization. For each outcome, GLMs incorporated three types of predictors: I. Variables significantly associated with the outcome in univariate analyses (*p* < 0.05); II. Key shift work demand variables (total night shift count, shift work hours, and shift workload exposure; total shift count was excluded due to multicollinearity) and circadian types (FR and LV); III. Potential confounders, including age, sex (5), and day workload exposure (years of work experience was excluded due to multicollinearity with age). Three candidate GLMs incorporating total night shift count, shift work hours, or shift workload exposure as moderators for PSQI and PHQ-9 scores were compared. Models were evaluated using the Akaike Information Criterion (AIC), Bayesian Information Criterion (BIC; log-likelihood based), and pseudo *R*^2^. Diagnostics included residual analysis and multicollinearity checks, with VIF < 5 considered acceptable.
(4) *Nonlinear Analysis*: To examine potential nonlinear associations, generalized additive models (GAMs) were applied to all predictors included in the optimal GLMs, with smooth terms for continuous variables and factor terms for categorical covariates (e.g., gender). Nonlinearity was evaluated based on effective degrees of freedom (EDoF) (EDoF substantially >1) and significance of smooth terms (*p* < 0.05). Subsequently, piecewise linear regression was used to identify potential breakpoints in shift work demand variables that showed significant nonlinear associations with the outcomes, and segment-specific slopes were compared to assess structural changes in these associations.
(5) *Monte Carlo Simulation*: In China, nurses predominantly work rotating shifts (both regular and irregular), while fixed or continuous night shifts are uncommon. In our study setting, nurses followed a predominant day–night (DN) rotation system, typically working one night shift (18:00–08:00) per week with a three-hour nap break (11 net working hours). To simulate real-world scenarios, predictive functions for PSQI and PHQ-9 scores were derived from the best-fitting GLMs using the corresponding original variables. A 100-day iterative simulation was performed, applying an exponentially weighted smoothing algorithm (*α* = 0.2), combining 20% of the current and 80% of the previous score to reduce short-term fluctuations. To assess robustness, alternative smoothing factors were tested in sensitivity analyses. Simulations were initialized with the minimum observed PSQI and PHQ-9 values in this dataset to represent a low-symptom baseline.

Two simulation strategies were adopted:

- *Population-based simulation*: Using empirical distributions from the 288-shift nurse sample, 1,000 virtual individuals were generated via bootstrap sampling. The trajectories of PSQI and PHQ-9 scores were simulated over 100 days to estimate population-level means and standard deviations.
- *Scenario-based group simulation*: Three circadian rhythm adaptability types were modeled by combining FR and LV scores: (a) Moderate adaptability (M): individuals with both FR and LV scores between the 25th and 75th percentiles; (b) High adaptability (H): FR score ≥75th percentile and LV score ≤25th percentile; and (c) Low adaptability (L): FR score ≤25th percentile and LV score ≥75th percentile. A typical 4-week shift work demands condition (Total night shift count = 4, Shift work hours = 44, Shift workload exposure median = 147) was set as the baseline and adjusted in sensitivity analyses. Each group comprised 1,000 simulated individuals tracked for 100 days. Their trajectories were compared to examine dose–response patterns under varying circadian type-shift work demand profiles.

## 3 Results

### 3.1 A total of 312 shift nurses were recruited

After excluding 24 participants (10 pregnant, 4 lactating, 2 with diabetes, 3 post-surgical, 2 with sickness absence >3 days and 3 without shift data), 288 nurses were included in the final analysis. Of these, 30 (10.42%) were male and 258 (89.58%) were female, with a median age of 33.5 years (range: 24–44). Regarding shift patterns, 188 nurses (65.28%) were engaged in day–night (DN) shifts, and 100 (34.72%) engaged in day-evening-night (APN) shifts. The median PSQI score was 8 (IQR: 4; range: 1–17), and the median PHQ-9 score was 7 (IQR: 5; range: 0–22). Based on classification criteria for insomnia and probable depression, 148 participants (51.39%) had insomnia, 70 (24.31%) had probable depression, and 58 (20.14%) had both conditions. Descriptive statistics of the variables are presented in Supplementary Table S1.

### 3.2 Univariate analysis

**Figure 1:**
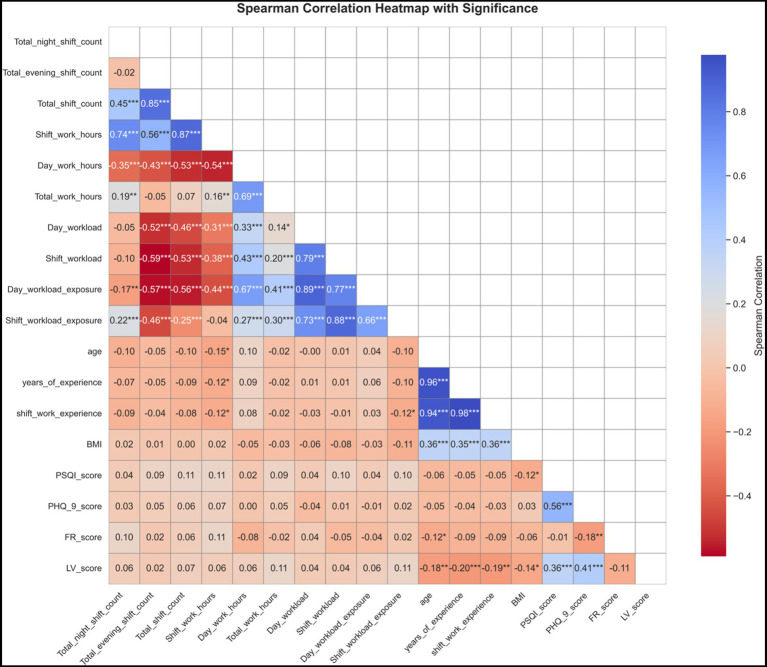
Heatmap of Spearman correlation coefficients among PSQI scores, PHQ-9 scores, FR scores, LV scores, and work demand related indicators. The color intensity in the figure reflects the strength of correlations between variables: darker blue indicates stronger positive correlations, while darker red indicates stronger negative correlations. PSQI, Pittsburgh Sleep Quality Index (Sleep quality); PHQ-9, Patient Health Questionnaire-9 (Depressive symptoms); FR, Flexible-rigid; LV, languid–Vigorous; BMI, Body Mass Index. Asterisks denote significance levels (***p < 0.001; **p < 0.01; *p < 0.05).

There were no statistically significant differences in sleep quality and depressive symptoms across sociodemographic groups (*p* > 0.05; Table 1). Spearman correlation analysis showed that poorer sleep quality (higher PSQI score) was positively correlated with depressive symptoms (*ρ* = 0.560, *p* < 0.001), greater languidness (*ρ* = 0.356, *p* < 0.001) and negatively correlated with body mass index (BMI) (*ρ* = −0.122, *p* = 0.038). Depressive symptoms were negatively correlated with greater flexibility (*ρ* = −0.179, *p* = 0.002) and positively correlated with greater languidness (*ρ* = 0.412, *p* < 0.001). Detailed results are shown in Figure 1 and Supplementary Tables S2a, S2b.

**Table 1:** Sociodemographic characteristics and relevant variables differences in PSQI, PHQ-9, FR, and LV scores (*N* = 288).

Variables	*N* (%)	PSQI median (IQR)	U or H statistic/*p* value	PHQ-9 median (IQR)	U or H statistic/*p* value	FR median (IQR)	U or H statistic/*p* value	LV median (IQR)	U or H statistic/*p* value
Gender									
Male	30 (10.4)	6.5 (3.8)	4437.0/0.188	8.0 (5.75)	3893.5/0.957	13.5 (6.0)	3438.0/0.316	17.5 (3.75)	**4801.5/0.030**
Female	258 (89.6)	8.0 (4.0)		7.0 (5.0)		12.0 (6.0)		19.0 (6.0)	
Age range									
20 ≤ years < 30	90 (31.3)	7.5 (4.0)	4.950/0.084	7.0 (4.0)	1.247/0.536	13.0 (5.0)	5.366/0.068	19.0 (6.75)	**10.735/0.005**
30 ≤ years < 40	167 (58.0)	8.0 (4.0)		7.0 (5.0)		12.0 (6.0)		18.0 (6.0)	
Years ≥ 40	31 (10.7)	6.0 (3.5)		6.0 (6.5)		13.5 (5.0)		17.0 (5.5)	
BMI									
BMI < 18.5	18 (6.3)	8.5 (3.5)	4.697/0.195	7.0 (2.75)	1.212/0.750	11.5 (5.75)	1.881/0.598	18.5 (3.75)	4.291/0.232
18.5 ≤ BMI < 24	218 (75.7)	8.0 (4.0)		7.0 (5.0)		13.0 (6.0)		19.0 (6.0)	
24 ≤ BMI < 28	48 (16.7)	7.0 (4.0)		7.0 (5.25)		12.0 (5.0)		17.5 (7.0)	
BMI ≥ 28	4 (1.4)	7.5 (4.5)		10.0 (4.75)		10.0 (3.5)		17.0 (1.0)	
Ethnicity									
Han	170 (59.0)	8.0 (4.0)	0.410/0.815	7.0 (5.75)	0.248/0.883	12.0 (5.0)	0.187/0.911	18.5 (6.0)	0.177/0.915
Zhuang minority	104 (36.1)	8.0 (4.0)		7.0 (5.0)		13.0 (6.0)		19.0 (5.0)	
Other	14 (4.9)	7.5 (3.75)		7.0 (5.5)		13.5 (8.5)		18.0 (4.25)	
Marital status									
Married	186 (64.6)	8.0 (4.0)	1.264/0.532	7.0 (5.0)	5.609/0.061	12.0 (5.75)	1.567/0.457	18.0 (5.0)	**8.293/0.016**
Unmarried	96 (33.3)	8.0 (4.0)		7.0 (4.0)		12.5 (6.0)		19.0 (6.25)	
Divorced	6 (2.1)	9.0 (2.75)		10.0 (0.75)		14.5 (4.0)		20.5 (6.0)	
Education level									
Associate degree	9 (3.1)	9.0 (2.0)	0.041/0.980	6.0 (4.0)	0.424/0.809	11.0 (5.0)	0.713/0.700	19.0 (5.0)	1.135/0.567
Bachelor’s degree	262 (91.0)	8.0 (4.0)		7.0 (5.0)		13.0 (6.0)		18.0 (6.0)	
Master’s degree or higher	17 (5.9)	7.0 (4.0)		6.0 (5.0)		12.0 (8.0)		20.0 (5.0)	
Work experience									
Years ≤ 5	71 (24.7)	7.0 (4.0)	3.645/0.456	7.0 (4.5)	0.806/0.938	14.0 (6.0)	3.668/0.453	19.0 (6.5)	**13.330/0.010**
5 < years ≤ 10	69 (24.0)	7.0 (4.0)		7.0 (5.0)		12.0 (6.0)		19.0 (6.0)	
10 < years ≤ 15	93 (32.3)	9.0 (4.0)		7.0 (4.0)		12.0 (6.0)		18.0 (5.0)	
15 < years ≤20	46 (16.0)	7.0 (3.75)		6.0 (7.0)		12.5 (4.5)		18.0 (4.75)	
Years > 20	9 (3.1)	6.0 (4.0)		8.0 (4.0)		15.0 (7.0)		16.0 (4.0)	
Professional title									
Junior and below	97 (33.7)	7.0 (4.0)	0.744/0.689	7.0 (4.0)	0.691/0.708	13.0 (5.0)	4.110/0.128	19.0 (7.0)	**9.102/0.011**
Intermediate grade	187 (64.9)	8.0 (4.0)		7.0 (6.0)		12.0 (6.0)		18.0 (5.0)	
Senior	4 (1.4)	9.0 (5.0)		6.0 (1.5)		14.0 (4.5)		14.0 (0.75)	
Number of children									
0	117 (40.6)	8.0 (4.0)	1.787/0.618	7.0 (5.0)	6.158/0.104	13.0 (6.0)	1.802/0.615	19.0 (7.0)	6.678/0.083
1	73 (25.3)	8.0 (4.0)		8.0 (5.0)		13.0 (7.0)		18.0 (5.0)	
2	96 (33.3)	7.0 (4.25)		6.0 (5.0)		12.0 (5.25)		18.0 (4.5)	
3	2 (0.7)	6.0 (3.0)		9.0 (3.0)		11.0 (1.0)		19.5 (5.5)	
Shift work experience									
Years ≤ 5	80 (27.8)	7.0 (4.0)	3.582/0.465	7.0 (4.25)	0.927/0.921	13.5 (6.5)	5.623/0.229	19.0 (6.0)	**14.614/0.006**
5 < years ≤ 10	86 (29.9)	8.0 (4.0)		7.0 (5.0)		12.0 (5.0)		18.0 (6.0)	
10 < years ≤ 15	76 (26.4)	8.0 (4.0)		7.0 (4.0)		13.0 (5.25)		19.0 (5.0)	
15 < years ≤ 20	40 (13.9)	7.0 (4.0)		6.0 (7.25)		13.0 (4.0)		17.0 (5.25)	
Years > 20	6 (2.1)	7.5 (3.25)		8.0 (3.75)		9.5 (6.25)		15.0 (3.5)	
Shift model									
DN	188 (65.3)	8 (5)	9315.5/0.900	7 (6)	9586.0/0.782	13 (6)	9722.0/0.632	19 (5.25)	9592.0/0.775
APN	100 (34.7)	8 (4)		7 (5)		12 (6)		18 (6.0)	

PSQI, Pittsburgh Sleep Quality Index (Sleep quality); PHQ-9, Patient Health Questionnaire-9 (Depressive symptoms); FR, Flexible-rigid; LV, languid–vigorous; IQR, Interquartile Range; U, Mann–Whitney U test; H, Kruskal-Wallis H test; BMI, Body Mass Index; DN, Day-Night shift; APN, Day (AM)-Evening (PM)-Night shift. Bold values indicate statistical significance (*p* < 0.05).

### 3.3 Predictors and moderators of sleep quality and depressive symptoms

All variables included in the GLMs had acceptable VIFs (range: 1.05–3.76, listed in Supplementary Table S3), indicating no serious multicollinearity. Residual analysis (residuals vs. fitted plots, Q–Q plots, Supplementary Figure S1) did not show major violations of model assumptions.

Among the three candidate models, the one including shift work hours as a moderator provided the best fit, with the lowest AIC (1377.37) and BIC (1428.05) and the highest pseudo *R*^2^ (0.4403), and was selected as the optimal GLM for sleep quality. Significant predictors of sleep quality included depressive symptoms (*β* = 0.245, 95% CI: 0.195–0.295), shift work hours (*β* = 0.093, 95% CI: 0.004–0.182), greater languidness (*β* = 0.065, 95% CI: 0.014–0.116), and BMI (*β* = −0.056, 95% CI: −0.105 to −0.007). The results indicated that greater depression severity, longer shift work hours, and greater languidness were associated with poorer sleep quality, whereas higher BMI showed a modest association with better sleep quality. Notably, the interaction terms of depressive symptoms with languidness (*p* = 0.090) and with shift work hours (*p* = 0.082) demonstrated marginal significance in predicting sleep quality. Details are shown in Table 2.

**Table 2:** Optimal generalized linear models predicting sleep quality and depressive symptoms among shift nurses.

Variables	*β*	Std err	*z*	*p*-value	95% CI
Sleep quality (PSQI score):					
Intercept	2.114	0.083	25.606	0.000	(1.953, 2.276)
Gender (female = 1)	−0.064	0.087	−0.736	0.462	(−0.234, 0.106)
PHQ-9 score	**0.245**	**0.025**	**9.637**	**0.000**	**(0.195, 0.295)**
Shift work hours	**0.093**	**0.046**	**2.047**	**0.041**	**(0.004, 0.182)**
PHQ-9 × shift work hours	−0.047	0.027	−1.741	0.082	(−0.101, 0.006)
LV score	**0.065**	**0.026**	**2.484**	**0.013**	**(0.014, 0.116)**
PHQ-9 × LV score	−0.035	0.021	−1.696	0.090	(−0.076, 0.005)
Shift work hours × LV score	0.014	0.026	0.548	0.584	(−0.036, 0.064)
Total night shift count	−0.050	0.039	−1.267	0.205	(−0.126, 0.027)
Shift workload exposure	0.002	0.041	0.052	0.958	(−0.078, 0.082)
Age	0.022	0.025	0.900	0.368	(−0.026, 0.071)
BMI	**−0.056**	**0.025**	**−2.237**	**0.025**	**(−0.105, −0.007)**
Daytime workload exposure	0.062	0.044	1.396	0.163	(−0.025, 0.148)
FR score	0.042	0.023	1.871	0.061	(−0.002, 0.086)
Depressive symptoms (PHQ-9 score):					
Intercept	1.809	0.109	16.535	0.000	(1.594, 2.023)
Gender (female = 1)	0.075	0.115	0.653	0.514	(−0.150, 0.300)
PSQI score	**0.314**	**0.034**	**9.122**	**0.000**	**(0.246,0.381)**
FR score	**−0.129**	**0.031**	**−4.171**	**0.000**	**(−0.190, −0.069)**
PSQI score × FR score	**0.091**	**0.028**	**3.212**	**0.001**	**(0.035, 0.146)**
LV score	**0.159**	**0.035**	**4.559**	**0.000**	**(0.090, 0.227)**
PSQI score × LV score	0.004	0.030	0.139	0.890	(−0.054, 0.062)
Shift work hours	−0.054	0.063	−0.848	0.396	(−0.178, 0.070)
PSQI score × shift work hours	−0.008	0.033	−0.230	0.818	(−0.072, 0.057)
FR score × shift work hours	−0.020	0.033	−0.599	0.549	(−0.084, 0.045)
LV score × shift work hours	**0.069**	**0.035**	**1.973**	**0.049**	**(0.000, 0.138)**
Age	0.009	0.032	0.268	0.789	(−0.054, 0.072)
Total night shift count	0.028	0.054	0.520	0.603	(−0.078, 0.135)
Shift workload exposure	0.008	0.057	0.133	0.894	(−0.104, 0.119)
Day workload exposure	−0.083	0.061	−1.347	0.178	(−0.203, 0.038)

PSQI, Pittsburgh Sleep Quality Index; PHQ-9, Patient Health Questionnaire-9; FR, Flexible-rigid; LV, languid–vigorous; CI, Confidence Interval. Bold values indicate statistical significance (*p* < 0.05).

Among the three candidate models, the one including shift work hours as a moderator provided the best fit, with the AIC (1657.89) and BIC (1712.20) and the highest pseudo *R*^2^ (0.4805), was selected as the optimal GLM for the depressive symptoms. Significant predictors of depressive symptoms included sleep quality (*β* = 0.314, 95% CI: 0.246–0.381), greater flexibility (*β* = −0.129, 95% CI: −0.190 to −0.069), greater languidness (*β* = 0.159, 95% CI: 0.090–0.227), and the interaction term of sleep quality with flexibility (*β* = 0.091, 95% CI: 0.035 ~ 0.146). The results indicated that poorer sleep quality and greater languidness were associated with more severe depressive symptoms, whereas greater flexibility was associated with lower depressive symptoms. The interaction between sleep quality and flexibility suggested that the protective association of flexibility was attenuated at higher levels of sleep disturbance. In addition, the interaction term of languidness with shift work hours showed marginal significance (*p* = 0.049). Details are shown in Table 2. Additional GLMs involving total night shift count and shift workload exposure as moderators are presented in Supplementary Table S4.

### 3.4 Nonlinear associations of shift work demand variables with sleep quality and depressive symptoms

**Figure 2:**
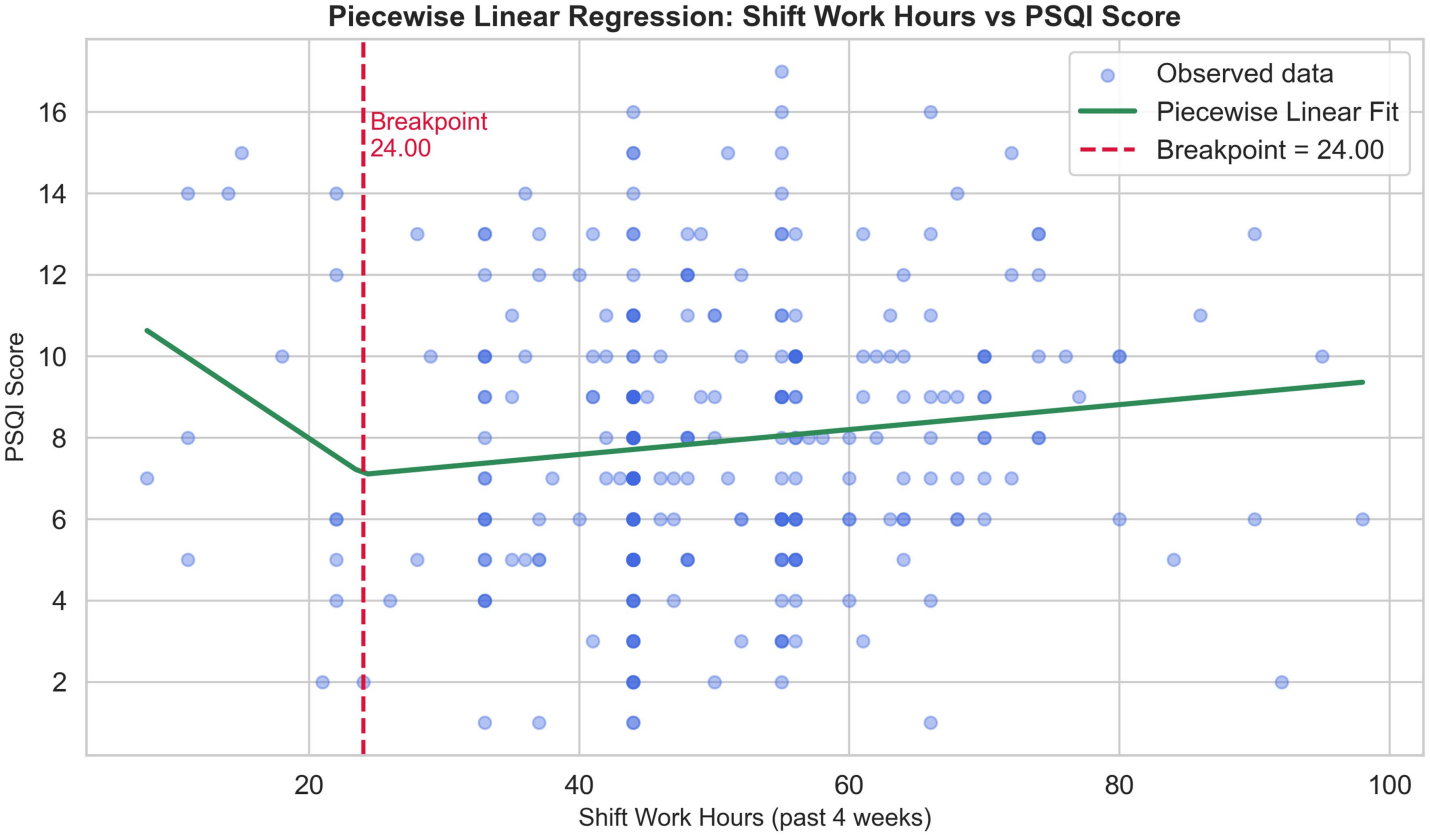
Piecewise linear relationship between shift work hours and sleep quality. A breakpoint was identified at approximately 24 shift work hours. Below this breakpoint, shift work hours were marginally negatively correlated with sleep quality (*β* = −0.221, *p* = 0.051), whereas above it, a significant positive correlation was observed (*β* = 0.031, *p* = 0.039). Green curves indicate the fitted segmented regression lines. PSQI, Pittsburgh Sleep Quality Index; Higher PSQI score indicates poorer sleep quality.

The GAM for sleep quality explained 40.5% of the variance (pseudo *R*^2^ = 0.405; GCV = 7.74; EDoF = 14.23). Among shift work demand variables, shift work hours showed a significant nonlinear association with sleep quality (EDoF = 1.6, *p* = 0.010), whereas night shift count (EDoF = 1.1, *p* = 0.189) and shift workload exposure (EDoF = 1.2, *p* = 0.623) did not. The GAM for depressive symptoms explained 44.1% of the variance (pseudo *R*^2^ = 0.441; GCV = 11.04; EDoF = 14.10), with no evidence of nonlinear effects for shift work hours (EDoF = 1.5, *p* = 0.691), night shift count (EDoF = 1.1, *p* = 0.548), or shift workload exposure (EDoF = 1.1, *p* = 0.830). Piecewise regression revealed a breakpoint at approximately 24 shift work hours per 4 weeks (Figure 2): below this threshold, no significant association with depressive symptoms (*β* = −0.221, *p* = 0.051) was observed, whereas above it, longer shift hours were significantly associated with poorer sleep quality (*β* = 0.031, *p* = 0.039).

### 3.5 Combined effects of circadian types and shift work demands

Sensitivity analyses showed that increasing the smoothing factor (*α* = 0.2 to 0.4) accelerated trajectories without altering overall dose–response patterns (Supplementary Figure S2), indicating robustness to smoothing assumptions, whereas sleep quality and depressive symptoms exhibited differential changes under varied shift work demands, reflecting sensitivity to shift work conditions.

**Figure 3:**
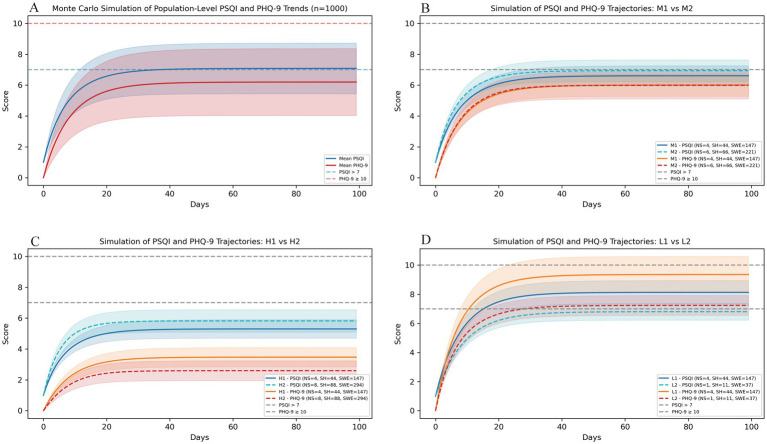
Monte Carlo simulation of sleep quality and depressive symptoms trajectories among shift nurses. **(A)** Simulated sleep quality and depressive symptoms trajectories over 100 days for 1,000 virtual nurses generated via bootstrap sampling from the study sample. Curves represent population-level mean trends and variability in sleep quality and depressive symptoms. **(B–D)** Simulated trajectories across three circadian rhythm adaptability profiles—moderate (M1/M2), high (H1/H2), and low (L1/L2)—under varying 4-week shift work demand conditions. In the moderate adaptability group, simulated trajectories indicated that exceeding roughly six night-shifts per 4 weeks could lead to a deterioration in sleep quality approaching the insomnia threshold **(B)**. Nurses with high adaptability maintained relatively favorable sleep and mood status even when shift work demands were doubled **(C)**. In contrast, those with low adaptability showed rapid deterioration in sleep quality and depressive symptoms under typical shift work conditions. Good sleep quality was maintained when shift work demands were reduced by approximately 75% **(D)**. PSQI, Pittsburgh Sleep Quality Index (Sleep quality); PHQ-9, Patient Health Questionnaire-9 (Depressive symptoms); FR, Flexible–Rigid; LV, languid–Vigorous.

The simulation suggested that the overall study population reached the clinical threshold for insomnia at approximately day 30, while maintaining a relatively good psychological status (Figure 3A). Under a typical 4-week shift work demand condition (total night shifts = 4, shift work hours = 44, shift workload exposure = 147), Individuals with moderate circadian adaptation could maintain healthy sleep quality and mental status. However, when the shift work demands increased by 50%, their sleep quality gradually declined and reached the clinical insomnia threshold (Figure 3B). Individuals with high circadian adaptability maintained favorable sleep quality and mental health even when shift work demands were doubled (Figure 3C). In contrast, individuals with low circadian adaptation showed a rapid deterioration in sleep quality and depressive symptoms under the typical shift work demand condition, surpassing the insomnia threshold by the second week. Their trajectories indicated that good sleep quality could only be maintained when shift work demands were reduced by approximately 75% (Figure 3D).

## 4 Discussion

This study, drawing on real-world shift work data and self-reported measures, explored the complex interrelationships among circadian rhythm types, shift work demands, sleep quality, and depressive symptoms in rotating-shift nurses. We found circadian rhythm types (FR/LV) and shift work hours exerted significant direct and/or moderating effects on sleep quality and depressive symptoms among shift-working nurses. Moreover, nonlinear associations and structural breakpoints were identified between sleep quality and shift work hours. Notably, nurses with different circadian rhythm types exhibited distinct dose–response patterns of sleep quality and depressive symptoms in response to shift work demands. These findings deepen our understanding of how circadian rhythm types (FR/LV) and shift work demands combine to influence sleep and depression in shift-working nurses. They also provide preliminary evidence to support personalized scheduling strategies and targeted health interventions.

In this study, we observed a bidirectional positive feedback relationship between sleep quality and depression: higher depression levels predicted poorer sleep quality (*β* = 0.245), and vice versa (*β* = 0.314). Although this reciprocal link has been well-established in prior research (42, 43), our findings notably suggest that the impact of poor sleep on depression is stronger than the reverse. This aligns with recent studies, such as Okechukwu et al., who identified poor sleep quality and quantity as key contributors to the mental health effects of night-shift work (44), and Brown et al. (45), who suggested that pronounced sleep disturbances often underlie the mental health consequences of shift work. Moreover, a prospective cohort study also demonstrated that insufficient or poor-quality sleep among shift workers is associated with a higher risk of depression (11), whereas a multicenter study found that adequate sleep is linked to lower stress levels (12). Together, these results underscore the foundational role of sleep disturbances in the development of depressive symptoms and support the prioritization of early sleep assessment and intervention in mental health strategies for shift-working nurses.

Circadian rhythm types emerged as significant predictors and moderators of poor sleep and depression. Greater languidness were associated with worse sleep quality (*β* = 0.065) and elevated depression levels (*β* = 0.159), while greater flexibility predicted lower depression levels (*β* = −0.129). Dynamic simulations further revealed distinct dose–response patterns across circadian profiles under shift work demands. These findings align with prior studies showing that greater languidness increases shift work disorder (SWD) risk (25, 27, 46). These associations may reflect that individuals with a languid trait have greater difficulty resisting drowsiness and require longer recovery from sleep loss, making them more vulnerable to sleep debt, poor sleep quality, and SWD after shift work. Conversely, greater circadian flexibility has been linked to better sleep (47) and lower SWD risk (46) among healthcare shift workers, although we did not observe a direct association between flexibility and sleep quality, which is consistent with previous reports (25, 27). This may reflect adaptive responses to chronic shift work or differences in sample characteristics. Overall, our results highlight the importance of circadian typology in identifying at-risk individuals and tailoring preventive interventions.

Research on the link between circadian rhythm types and depression remains limited, though some studies suggest associations between chronotype alterations and depressive symptom severity (48). Another study (49) also found that circadian rhythm types significantly moderated the relationship between perceived stress and sleep quality. To the best of our knowledge, this study is among the first to integrate objective shift workload data and demonstrate that circadian rhythm types directly predict depression levels and that flexibility moderates the impact of poor sleep quality on depression. Greater flexibility were linked to a protective effect, with lower depression levels, however, the PSQI × FR interaction showed that this protective link became weaker as sleep quality worsened. This may reflect that greater flexibility, although facilitating adaptation to irregular schedules, may not fully counteract the adverse consequences of circadian misalignment and melatonin disruption when sleep quality deteriorates, thereby intensifying the emotional impact of poor sleep. Collectively, these findings suggest that circadian traits may play an important role in shaping sleep–emotion dynamics and indicate the need for further investigation into their complex interactions.

Shift work demands were a primary focus of this study. We found that shift work hours had a direct impact on sleep quality (*β* = 0.093) and positively moderated the association between LV and depression (*β* = 0.069). These findings are consistent with a recent 4.5-year prospective cohort study that identified a dose–response pattern between shift work and sleep disorders and recommended that night shifts should not exceed 50 within a six-month period (50). However, we did not observe a significant association between total night shift count and sleep or depression. By contrast, a study of Korean shift nurses also reported a positive correlation between monthly night shift frequency and insomnia risk (52). Similarly, a prospective cohort study based on the UK Biobank (53) found that shift workers with more than eight night-shifts per month had an increased risk of depression (HR = 1.40). This discrepancy may reflect the complex relationship between total night shifts and health outcomes, underscoring the need for threshold-based or dose–response modeling.

We further observed dose–response relationships between shift work hours and sleep quality using nonlinear modeling and breakpoint analysis. Notably, when shift work hours exceeded about 24 h within a four-week period, sleep quality appeared to deteriorate progressively with increasing shift hours. These findings align with previous studies reporting that longer working hours and more working days are associated with greater sleep disturbances and poorer sleep quality among shift nurses (21, 51). Taken together, our findings suggest that shift work hours may represent meaningful “exposure doses” of shift burden. These cumulative doses, specifically when shift work hours exceed 24 in 4 weeks, warrant close attention from nursing administrators. Dynamic simulation further revealed distinct dose–response patterns across circadian adaptability profiles. Nurses with moderate adaptability tolerated typical shift work demands, whereas those with low adaptability showed more rapid deterioration in sleep and mood. For individuals with low adaptability, the simulations suggested that a workload of about one night shift per month might be tolerable; however, this requires confirmation in controlled trials. These scenario-based findings imply that the importance of considering both shift work demands and individual circadian traits when developing personalized scheduling strategies and targeted health interventions for nurses.

## 5 Conclusion

This study highlights the significant direct and moderating effects of circadian rhythm types and shift work hours on sleep quality and depressive symptoms among shift-working nurses. Importantly, a potential threshold effect was observed, with the risk of poor sleep quality appearing to increase when cumulative shift hours exceeded approximately 24 h within a four-week period. The combined effects of circadian rhythm types and shift work demands corresponded to distinct dose–response patterns in sleep quality and depressive symptoms. These findings deepen our understanding of the mechanisms underlying sleep and mental health in shift-working nurses and offer preliminary evidence to support personalized scheduling strategies and targeted health interventions.

### 5.1 Limitations

Several limitations should be noted. First, the cross-sectional design limits causal inference; longitudinal or cohort studies are needed to clarify the directionality of associations. Second, due to constraints in accessing objective work demand data, participants were recruited from a single center and were predominantly female (89.58%), which may limit generalizability, particularly to male shift workers. Third, a baseline health check was not conducted prior to the study, which may have introduced bias, as some pre-existing depressive symptoms could have been overlooked, although participants with major psychiatric disorders were excluded. Fourth, work demands were assessed over a four-week period; future studies should extend the observation window (e.g., 12–24 weeks) to capture longer-term effects. Moreover, simulations were based on relatively stable shift patterns and did not account for daily scheduling variations, limiting their ability to model the dynamic accumulation and recovery processes. Finally, sleep and depressive symptoms were measured using self-reports, which may be subject to recall or reporting bias. Incorporating objective assessments in future research is warranted to improve measurement precision.

## Data Availability

The original contributions presented in the study are included in the article/Supplementary material, further inquiries can be directed to the corresponding authors.
